# IRES-mediated *Pichia pastoris* cell-free protein synthesis

**DOI:** 10.1186/s40643-023-00653-4

**Published:** 2023-06-20

**Authors:** Yanan Wang, Ting Wang, Xinjie Chen, Yuan Lu

**Affiliations:** 1 Key Laboratory of Industrial Biocatalysis, Ministry of Education, Tsinghua University, Beijing, 100084 China; 2 Department of Chemical Engineering, Tsinghua University, Beijing, 100084 China

**Keywords:** *Pichia pastoris*, Cell-free protein synthesis, In vitro transcription–translation, IRES

## Abstract

Cell-free protein synthesis (CFPS) system is an ideal platform for fast and convenient protein research and has been used for macromolecular assembly, unnatural amino acid embedding, glycoprotein production, and more. To realize the construction of an efficient eukaryotic CFPS platform with the advantages of low cost and short time, a CFPS system based on the yeast *Pichia pastoris* was built in this study. The internal ribosomal entry site (IRES) can independently initiate translation and thus promote protein synthesis. The Kozak sequences can facilitate translation initiation. Therefore, the screening of IRES and its combination with Kozak was performed, in which cricket paralysis virus (CRPV) exhibited as the best translation initiation element from 14 different IRESs. Furthermore, the system components and reaction environment were explored. The protein yield was nearly doubled by the addition of RNase inhibitor. The cell extract amount, energy regeneration system (phosphocreatine and phosphocreatine kinase), and metal ions (K^+^ and Mg^2+^) were optimized to achieve the best protein synthesis yield. This *P. pastoris* CFPS system can extend the eukaryotic CFPS platform, providing an enabling technology for fast prototyping design and functional protein synthesis.

**Graphical Abstract:**

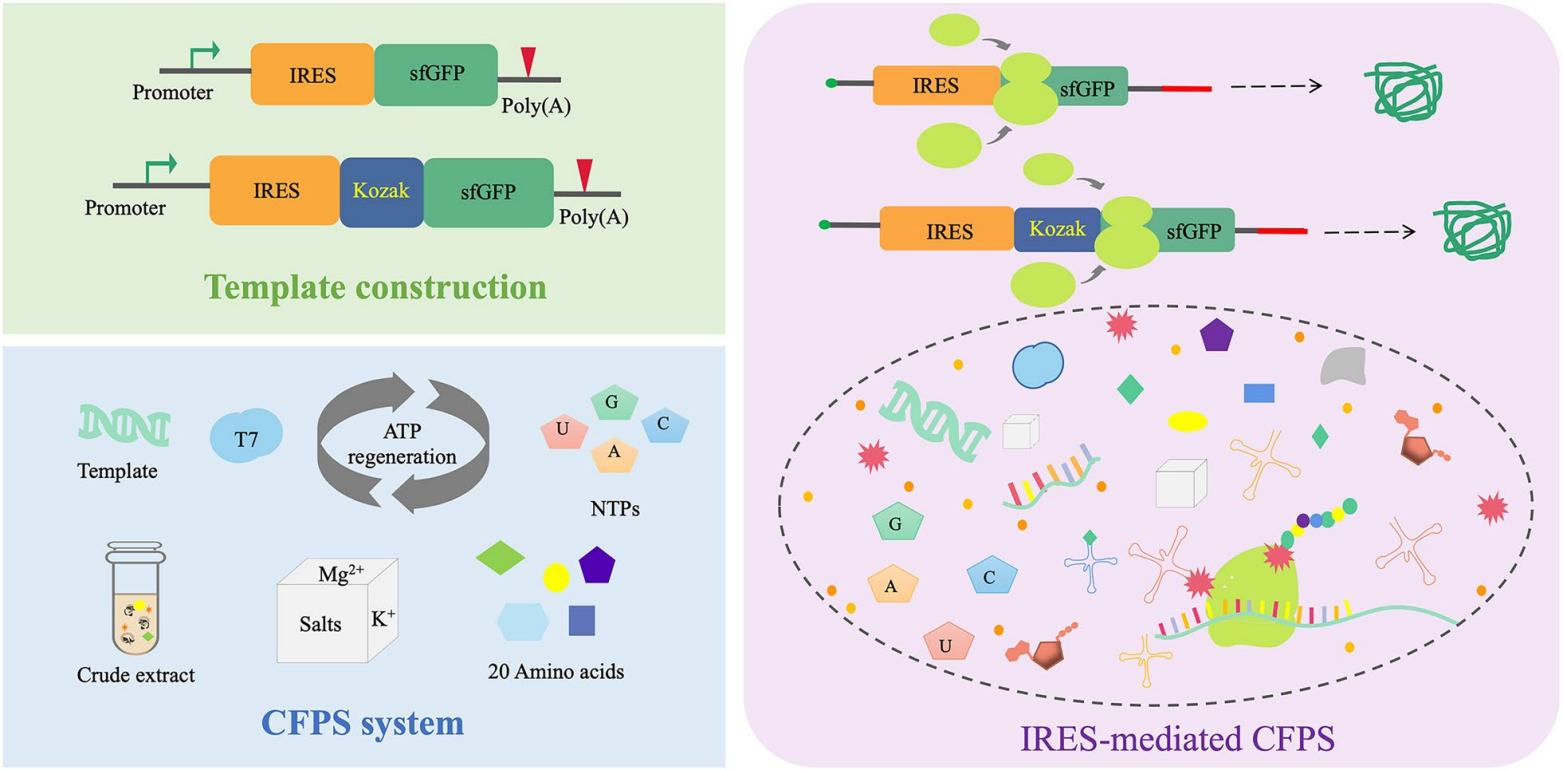

**Supplementary Information:**

The online version contains supplementary material available at 10.1186/s40643-023-00653-4.

## Introduction

Advances in synthetic biology are driving the development of cell-free protein synthesis (CFPS), which is emerging as a transformative biomanufacturing platform. CFPS is an in vitro transcription–translation system that synthesizes proteins by exogenous addition of genetic templates, enzymes, substrates, and energy (Wang et al. 2022a). The crude extract contains biological components required for translation, protein folding, and energy regeneration (Li et al. 2022). The rapid development of CFPS is mainly due to its potential for rapid expression of biologically active recombinant proteins. Compared to in vivo synthesis, the CFPS system does not require concerns about transmembrane transport and cytotoxicity, and all the energy in the system can be used to produce the protein of interest (Hou et al. 2022), which can greatly increase the efficiency of protein expression. It is also possible to use linear DNA as the expression template, avoiding the time-consuming cloning step of plasmid-based methods, giving the CFPS system the potential to study, screen and engineer proteins in a high-throughput manner (Chiba et al. 2021). Currently, CFPS systems have been applied to unnatural amino acid embedding (Wang et al. 2022b), protein structure analysis (Alfi et al. 2022), synthesis of membrane proteins (Mezhyrova et al. 2022), synthesis of glycoproteins, and drug development (Dondapati et al. 2020). Therefore, the CFPS system has been a cutting-edge and hot topic in bioengineering research.

Depending on the source of the crude extract, CFPS can be divided into two main types: prokaryotic and eukaryotic. The common prokaryotic crude extract systems include *Escherichia coli*, *Bacillus subtilis*, and *Corynebacterium glutamicum* (Smolskaya et al. 2020). Prokaryotic CFPS has the advantages of high batch yield and low cost, and has been commercialized as a genetic tool for synthetic biology studies (Zhang et al. 2020). However, a key issue with prokaryotic CFPS is the post-translational modification limitation (Perez et al. 2016). Common eukaryotic crude extract systems include *Saccharomyces cerevisiae* (Kim et al. 2020), tobacco BY-2 (Buntru et al. 2022), Chinese hamster ovary cells (Thoring et al. 2017), wheat germ (Kanoi et al. 2021), and insects (Imamura et al. 2019). However, these well-established CFPS systems have their own disadvantages, such as extract preparation being long and labor intensive, difficult technology transfer, and low protein yields (Zemella et al. 2015). Currently, most eukaryotic CFPS systems are costly and time-consuming and cannot be mass-produced (Gregorio et al. 2019). Therefore, this study is very much interested in developing less costly and time-consuming CFPS systems based on eukaryotic microorganisms to expand the protein expression toolkit for rapid protein synthesis, vaccine development, and macromolecular assembly.

Currently, the yeast chassis for establishing CFPS systems mainly include *S. cerevisiae* and *Pichia pastoris*. *S. cerevisiae* is the model strain and has been commonly used in studies, such as metabolic engineering. Although *S. cerevisiae* can be used in CFPS systems, it still has problems, such as low protein synthesis ability. However, *P. pastoris* is more commonly used for protein expression, which could be a better CFPS chassis than *S. cerevisiae*. *P. pastoris* is gaining more and more attention as an efficient expression system that is widely used in gene expression studies.* P. pastoris* is commonly used as a safe microorganism to express some toxic proteins and easily degraded enzymes (Yang and Zhang 2018), and are widely used for protein production (Aw et al. 2020). In recent years, due to its ability to grow rapidly on simple media (Karbalaei et al. 2020) and its well-defined genome sequence (Juturu and Wu. 2018), *P. pastoris* has become an attractive CFPS chassis that noticed by researchers (Rinnofner et al. 2022), but the protein synthesis yield of cell extracts prepared from this strain still needs to be improved (Wang et al. 2020; Zheng et al. 2019). Internal ribosome entry sites (IRES) are RNA elements that recruit ribosomes to the internal region of mRNA to initiate translation independently (Yang and Wang 2019). The study of IRES sequences is of great importance for gene expression (Kim and Siddiqui 2021). It has been shown that IRESs in eukaryotic cells initiate translation independently in two ways, either IRES trans-acting factor (ITAFs) bound to cis elements interact with ribosomes, or IRESs contain a short cis element that pairs with 18 S rRNA to recruit ribosomes (Dresios et al. 2006; Yang et al. 2017). The Kozak sequence is adjacent to the ATG initiation codon, which greatly improves the translation efficiency and the overall expression of the gene product. It works by slowing the scanning rate of the ribosome and increasing the probability of identifying the translation initiation of the AUG initiation codon (Olafsdóttir et al. 2008). Therefore, combining IRES and Kozak with the* P. pastoris* CFPS system might promote efficient protein expression.

**Fig. 1:**
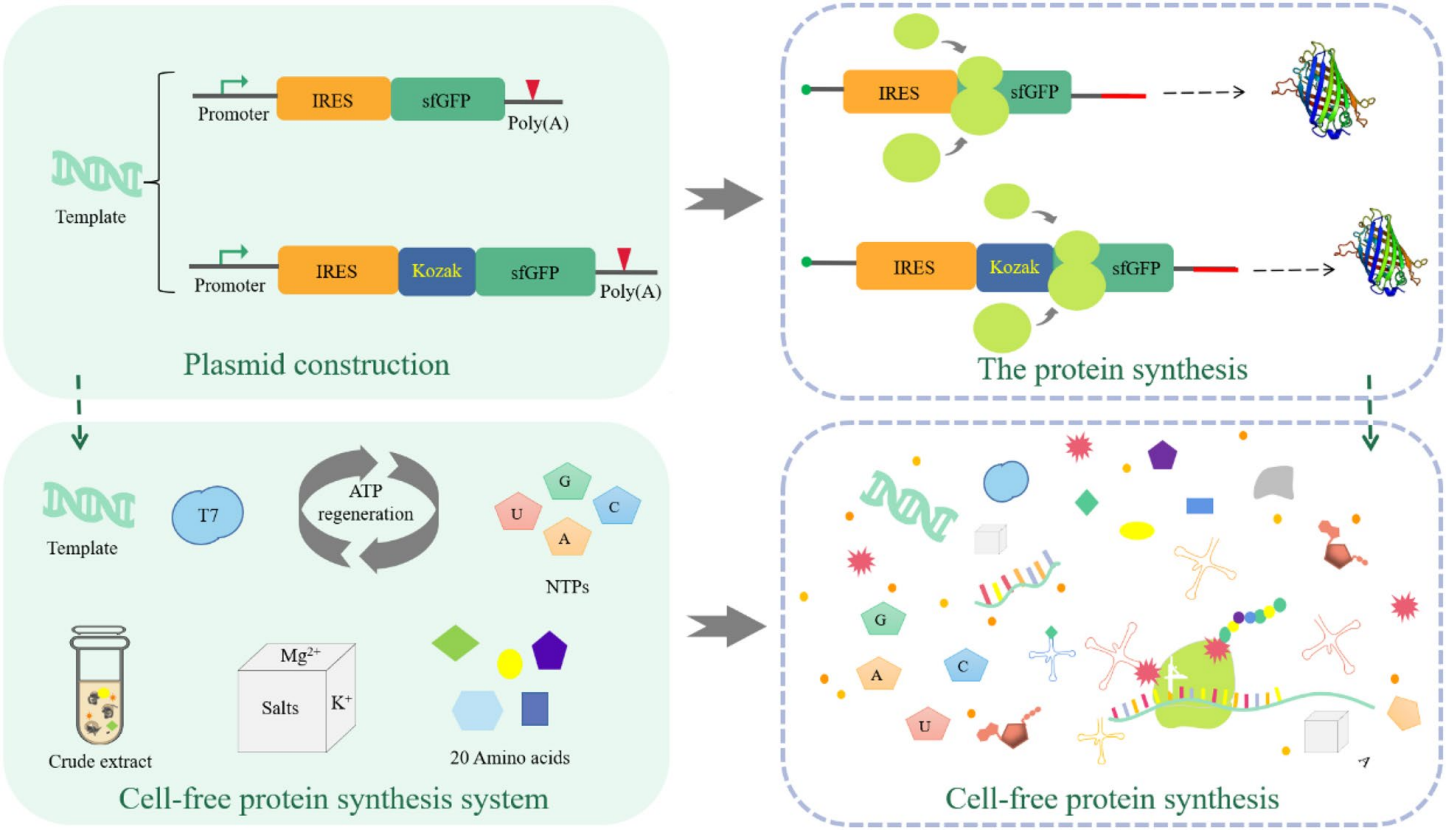
IRES-mediated platform for efficient *P. pastoris* cell-free protein synthesis. IRES and Kozak sequences were introduced to promote protein synthesis in the CFPS system. Using this design principle, the protein expression was regulated at the translation level

In this study, the strain *P. pastoris* SMD1163 was used to build an efficient eukaryotic CFPS system. Then, using the constructed *P. pastoris* platform, IRES and Kozak sequences were screened to promote protein translation to achieve better protein synthesis in the cell-free system. Finally, the energy supply, the time trend of protein synthesis, and the effect of metal ions were explored to further promote efficient CFPS. It would provide more possibilities to further improve the translation efficiency of eukaryotic CFPS for broader applications (Fig.1).

## Results and discussion

### Establishment of pichia pastoris CFPS system and IRES screening

*P. pastoris* has the advantages of rapid growth on simple media (Karbalaei et al. 2020) and well-defined genome sequence (Juturu and Wu 2018), commonly used for the expression of some toxic proteins and easily degraded enzymes (Yang and Zhang 2018), which has been widely used for protein production (Aw et al. 2020). *P. pastoris* is becoming an attractive chassis for the construction of CFPS systems (Rinnofner et al. 2022).

**Fig. 2:**
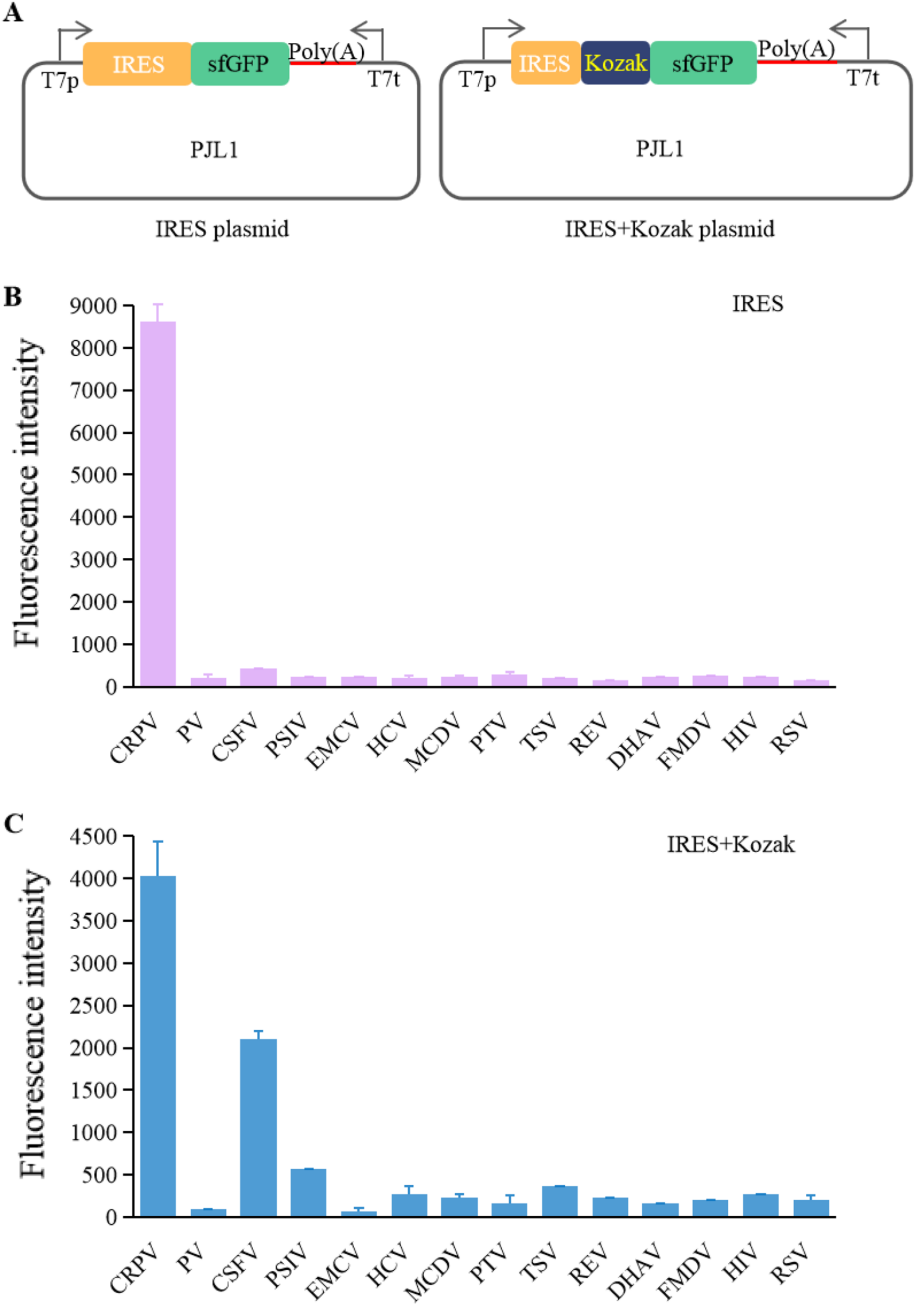
IRES screening of *P. pastoris* CFPS. **A** Schematic diagram of IRES plasmid construction. **B** CFPS with different IRES constructs. **C** CFPS by integrating different IRESs with Kozak sequence. The values in the graph are the mean values of three independent experiments, and the error line represents the standard deviation. *CRPV* Cricket paralysis virus, *PV* Poliovirus, *CSFV* Classical swine fever virus, *PSIV* Plautia stali intestine virus, *EMCV* Encephalomyocarditis virus, *HCV* Hepatitis C virus, *MCDV* Mud crab dicistrovirus, *PTV* Porcine teschovirus, *TSV* Taura syndrome virus, *REV* Reticuloendotheliosis virus, *DHAV* Duck hepatitis A virus, *FMDV* Foot-and-mouth disease virus, *HIV* Human immunodeficiency virus, *RSV* Rous sarcoma virus

With the study of the IRES mechanism, it is gradually understood that IRES recruits ribosomes through ITAFs or a short cis element to independently initiate translation and enhance the regulation of downstream gene expression. To enhance protein expression, an IRES-mediated *P. pastoris* SMD1163 CFPS platform was constructed. Fourteen common IRES sequences (Additional file 1: Table S2) that could potentially initiate translation independently were used for the screening (Fig. 2A). These IRES sequences are all active in their natural structure. The IRES sequences chosen for the experiments are those that were discovered early. IRES-mediated translation results showed that CRPV had a high expression level, but the rest IRESs had low expression levels (Fig. 2B). The activities of IRESs often require the assistance of ITAFs, but CRPV can assemble the translation initiation complex by binding to the 40 S subunit without ITAFs and initiation factors, and then directly ligate to the 60S subunit to produce an 80 S ribosome with elongation capacity (Pestova et al. 2004). Maybe this was the reason why CRPV showed better expression ability than other IRESs.

Besides IRES, Kozak sequence can promote the translation initiation of mRNA with 5′-end cap structure by combining it with the translation initiation factor. Therefore, to better promote translation initiation, Kozak sequence (GAAACG) was added after the IRES sequence for screening (Fig. 2A). As shown in Fig. 2C, the simultaneous translation initiation by IRES and Kozak revealed that CRPV and CSFV successfully achieved protein expression, with the expression of CSFV slightly lower than that of CRPV, and the rest IRESs had lower expression. The decrease in CRPV protein expression yield after the addition of Kozak sequence may be due to the fact that the secondary structure of RNA without Kozak has a Gibbs free energy of − 429.09 kcal/mol, whereas the secondary structure of RNA with Kozak has a Gibbs free energy of − 428.79 kcal/mol. The change in the secondary structure affected the protein translation process. Compared with no Kozak sequence addition, the protein expression of CSFV-mediated protein was significantly higher after the addition. It has been shown that the Kozak sequence was often used to construct expression vectors before the start codon to enhance protein translation and recombinant protein yield (Várnai et al. 2014). Therefore, the CRPV with the highest protein expression as well as Kozak, was used for subsequent optimization.

### CFPS system exploration

**Fig. 3:**
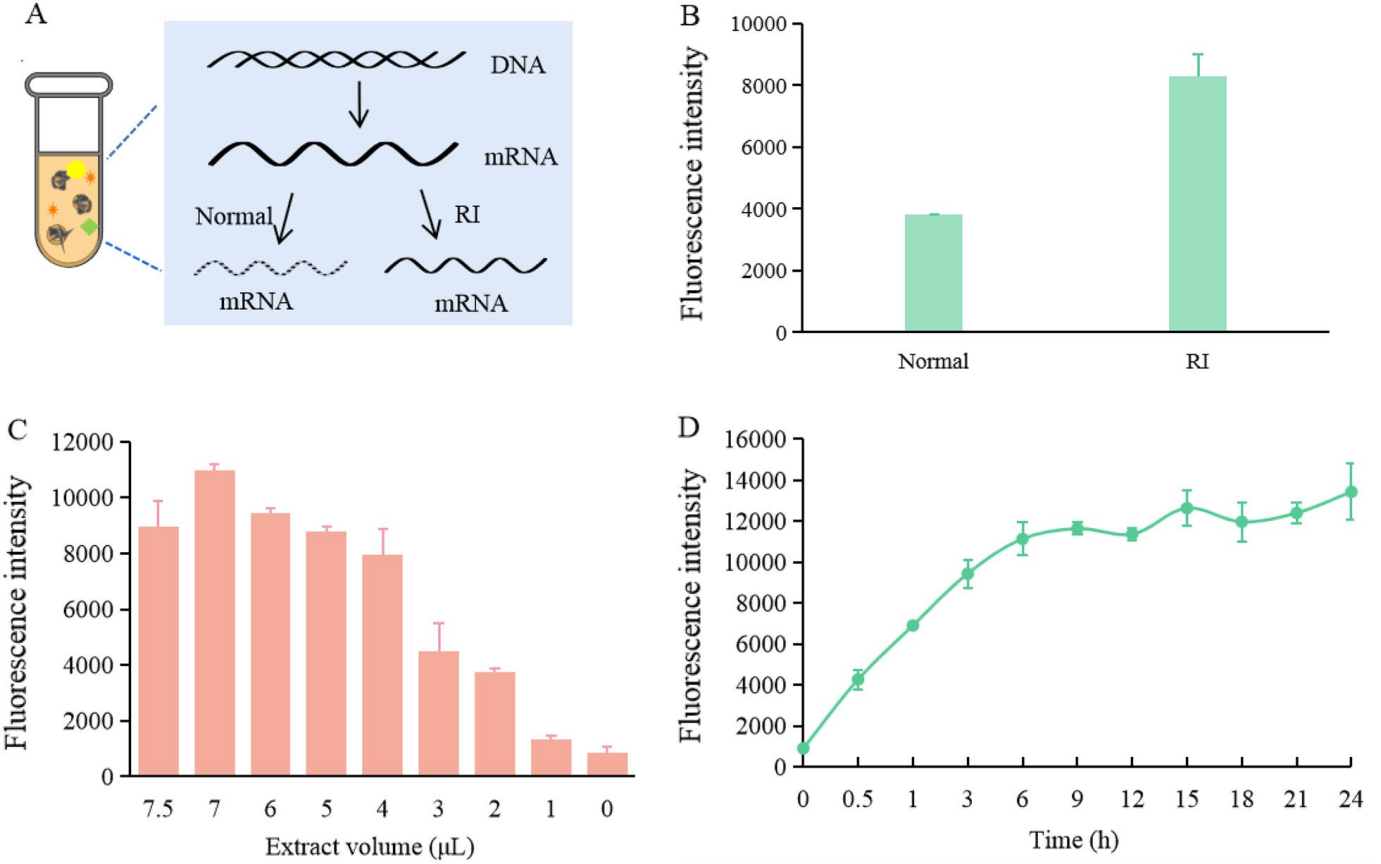
Transcription optimization, screening of extract contents, and investigation of reaction time. **A** Schematic diagram of RI promotion for yield enhancement. **B** Effects of RI addition on protein expression. **C** Optimization of the extract volume. **D** Investigation of CFPS reaction time. The values in the graph are the mean values of three independent experiments, and the error line represents the standard deviation

There is an inevitable problem of exogenous RNase contamination during the preparation of extracts and cell-free transcription and translation experiments. It leads to the degradation of mRNA, which further reduces the protein yield. RNase inhibitor (RI) may prevent RNase from attacking and destroying mRNA, which could slow down the degradation rate of mRNA. Therefore, it is meaningful to add RI to improve the final yield of the system (Fig. 3A). The RNase inhibitor is an recombinantly expressed human placental RNase inhibitor. RI addition was 20 U per individual reaction system. Figure 3B shows that the sfGFP yield of the system was significantly increased by adding RI, which was twice as high as the yield without optimization. It has been shown that RNase can degrade RNA (Marshall et al. 2008), while RI can bind to heterologous RNase (Johnson et al. 2007). Therefore, the addition of RI could be an effective strategy to improve the *P. pastoris* CFPS.

As the core of the CFPS system, cell extract contains initiation factors, elongation factors, and release factors needed for protein synthesis. The amount of extract affects the content of these factors in the system. Therefore, the addition volume of extracts was next screened. The results showed (Fig. 3C) that the yield gradually increased with increasing extract volume, reached the highest sfGFP yield at 46% (v/v), and showed a high and stable yield at 33% (v/v). To ensure that subsequent optimization could provide a reasonable space, 33% (v/v) extract volume was used for all subsequent fraction optimization.

The reaction time determines the rate of protein synthesis, so it is necessary to select the optimal reaction time and explore the changing trend of the system to maximize the yield. The results of the time investigation showed (Fig. 3D) that the rate of sfGFP synthesis was highest in the first 1 h and increased almost linearly. The rate of protein synthesis decreased from 1 to 6 h, and there was no significant increase in fluorescence after 6 h. Therefore, the optimal time to achieve high yield with minimum time consumption was determined to be 6 h. Previous eukaryotic CFPS studies have shown that the reaction time for tobacco CFPS takes 48 h and 12 h for CHO CFPS (Das Gupta et al. 2022; Heide et al. 2021). Thus, the *P. pastoris* system has a shorter reaction time compared to other eukaryotic CFPS systems.

### Energy optimization

**Fig. 4:**
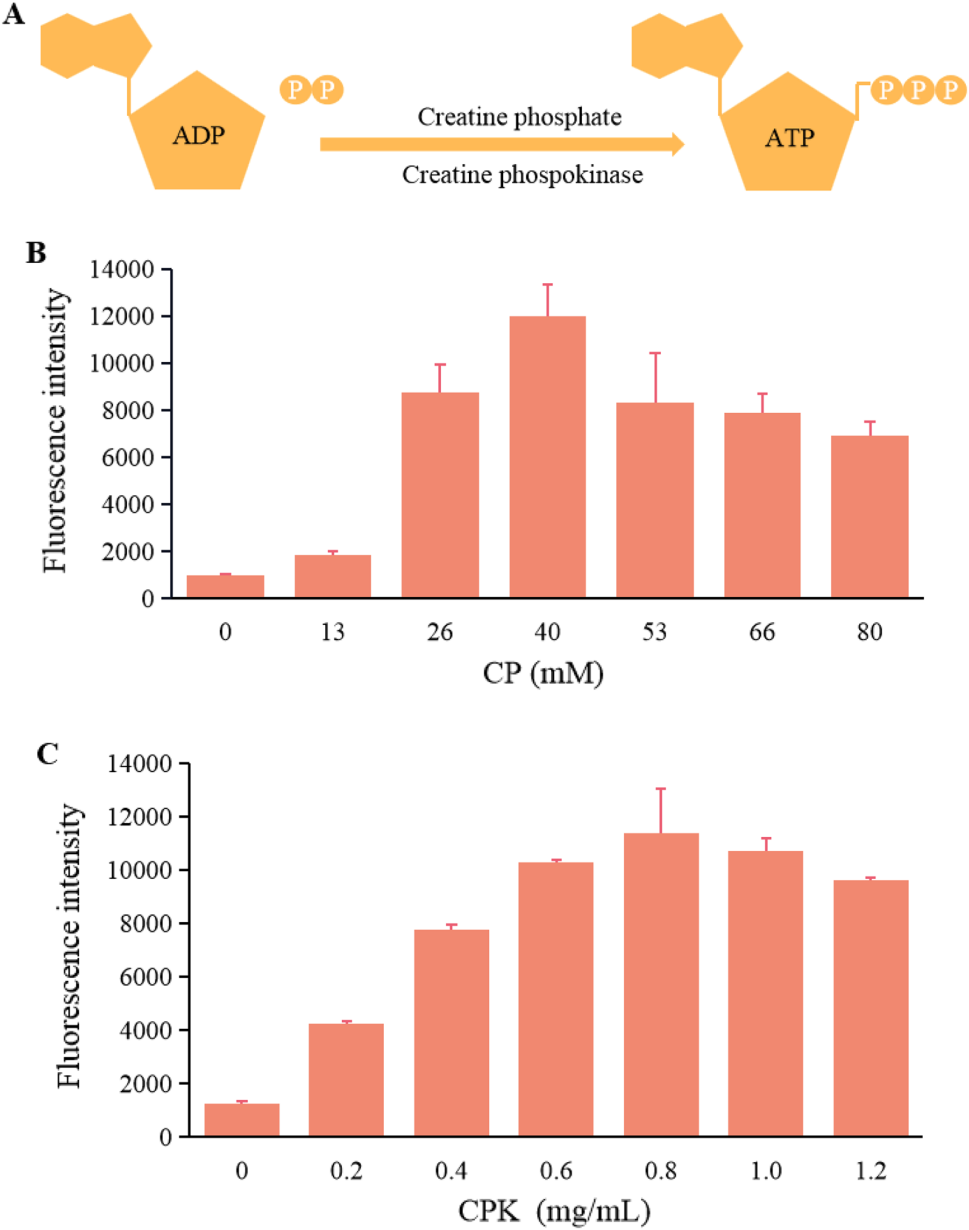
Energy generation and component optimization. **A** Schematic diagram of energy generation. **B** CP concentration optimization. **C** CPK concentration optimization. The values in the graph are the mean values of three independent experiments, and the error line represents the standard deviation

Ribosome movement, tRNA transport of amino acids, and other life activities require energy. ATP, as the energy compound, is closely related to protein transcription and translation, affecting the efficiency of protein synthesis. Therefore, energy supply is important for developing a functional *P. pastoris* CFPS platform. As shown in Fig. 4A, energy production in eukaryotic cells uses ADP and phosphocreatine as substrates to be converted to ATP via phosphocreatine kinase. Phosphocreatine/phosphocreatine kinases (CP/CPK) are often used for energy supply in eukaryotic CFPS systems, because there is no other metabolic pathway for the synthesis and breakdown of phosphocreatine in cells. Therefore, the energy regeneration system based on CP/CPK was optimized. Figure 4B shows that the yield gradually increased with increasing CP concentration, reaching the highest yield at 8% (v/v), after which the yield gradually decreased with increasing concentration. The results of CPK optimization are shown in Fig. 4C. With the highest yield at 21% (v/v) of CPK volume, after that, the yield decreased slowly. Some studies have shown that a stable energy supply can ensure the continuity of the reaction (Calhoun and Swartz. 2007). However, adding too much CPP/CPK also could inhibit the read-through of UGA codon and affect the termination of translation (Ahn et al. 2005), so the protein yield could be affected when it is too high or too low. In summary, the optimal energy generation-related volume ratios were determined to be 8% CP and 21% CPK.

### CFPS reaction condition exploration

**Fig. 5:**
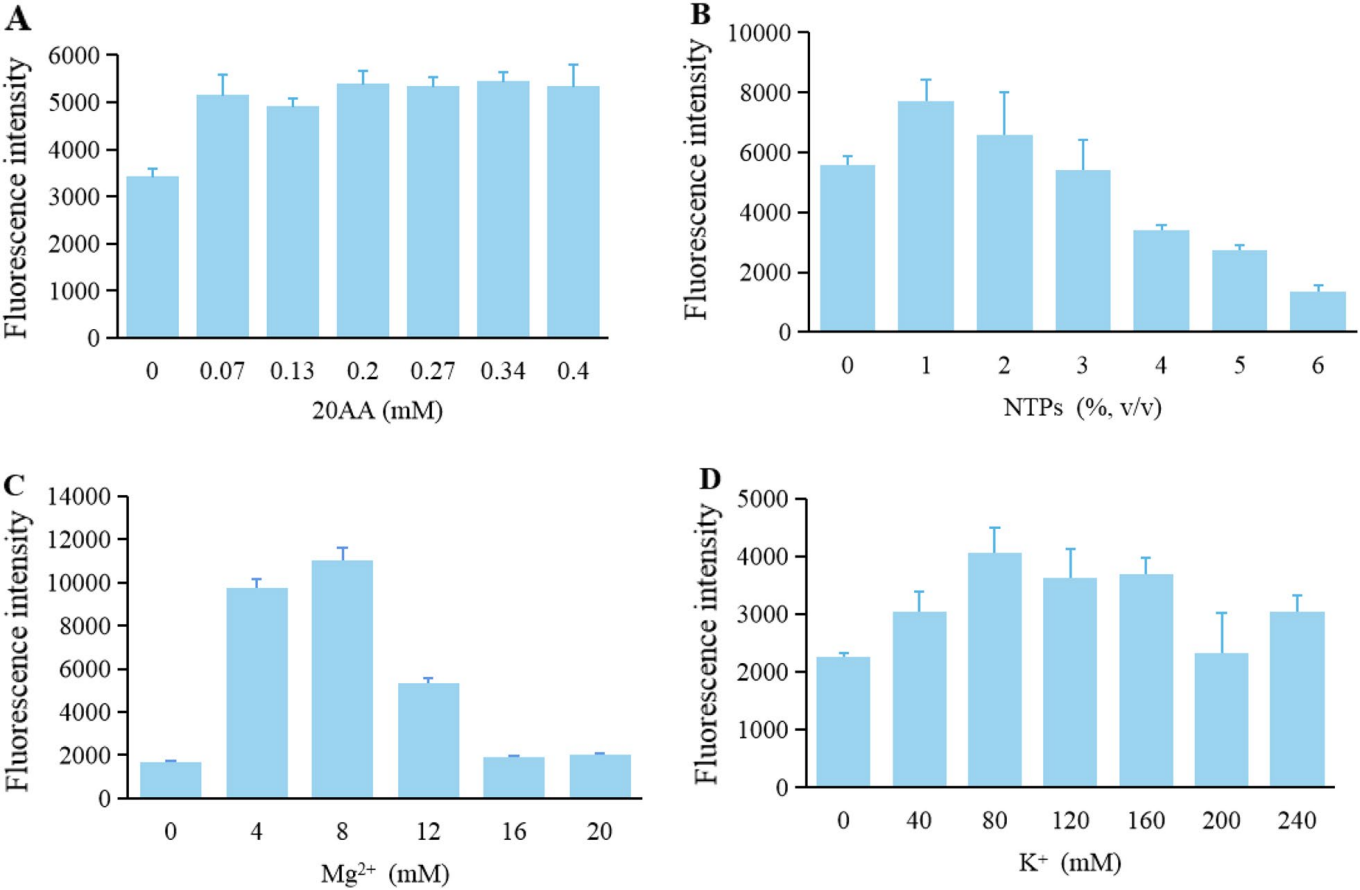
Optimization of CFPS reaction conditions. **A** 20AA concentration optimization. **B** NTPs addition optimization. **C** Mg^2+^ concentration optimization. **D** K^+^ concentration optimization. The values in the graph are the mean values of three independent experiments, and the error line represents the standard deviation

As the basic unit of protein, amino acid is an indispensable substrate in protein synthesis. Therefore, a stable supply of amino acids is very important, and its volume needs to be optimized. Figure 5A shows that when the volume of amino acids was above 0.07 mM, the yield of sfGFP was basically stable. When there was no amino acid addition, some proteins still could be produced. Studies have shown that in the preparation of crude cell extract, the metabolism is still retained (Vilkhovoy et al. 2020), which causes the residues of amino acids, promoting the expression of a small amount of protein.

NTPs mixture in the CFPS system include the basic unit of RNA and components participating in the transcription of DNA. Therefore, the volume optimization of NTPs was carried out. As shown in Fig. 5B, the results presented that the protein synthesis yield was the best when the volume of NTPs was 1%, and the protein yield decreased gradually with the increase of NTPs volume. Takahashi et al*.* also found that higher concentration of NTPs inhibited protein synthesis in the *E. coli* CFPS (Takahashi et al. 2021), which was consistent with the experimental results of *P. pastoris* CFPS system.

Metal ions can greatly affect the transcription and translation of CFPS system. K^+^ can participate in the binding of tRNA to ribosome, and Mg^2+^ can catalyze, activate and participate in the catalytic reaction. During the screening of Mg^2+^ concentration, the concentration of K^+^ was 180 mM. The concentration of Mg^2+^ during the screening of K^+^ was 4 mM. K^+^ optimization results showed (Fig. 5C) that the best protein yield was achieved at a concentration of 80 mM, and the yield decreased significantly when the concentration reached 200 mM. Mg^2+^ optimization results showed (Fig. 5D) that the highest protein yield was achieved at a concentration of 8 mM, and then decreased significantly. Usually, different CFPS systems require different concentrations of metal ions (Kelwick et al. 2016; Lin et al. 2019), more metal ions might be toxic to CFPS. Therefore, the optimal metal ion concentrations in the *P. pastoris* CFPS system were 80 mM for K^+^ and 8 mM for Mg^2+^.

**Fig. 6:**
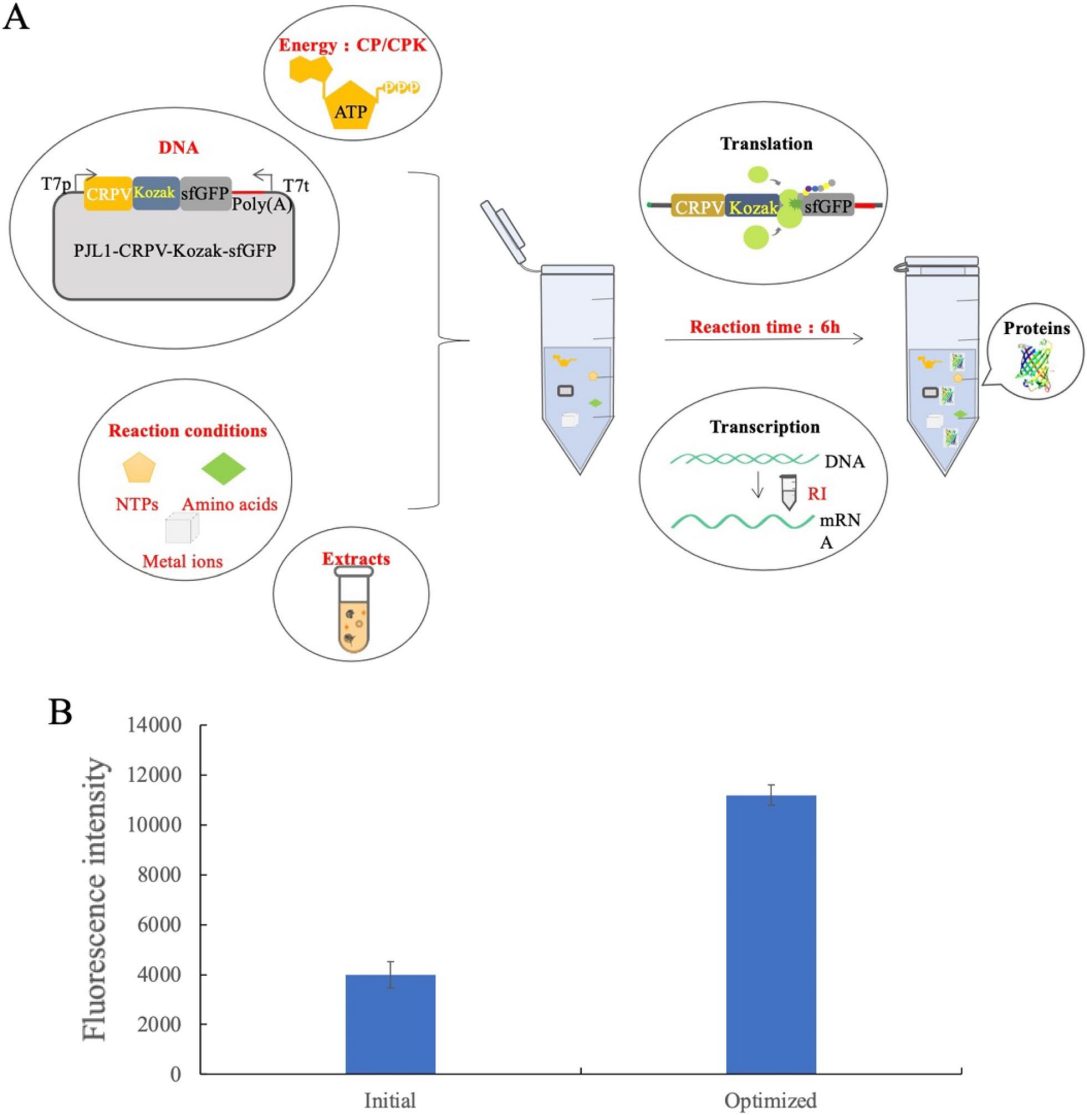
*P. pastoris* CFPS after design and optimization. **A** *P. pastoris* CFPS after the design and optimization. The components of the CFPS system, including extracts, DNA, energy, and reaction conditions, were optimized, and the protein synthesis reactions were promoted from two levels of transcription and translation. The red font in the figure shows the various factors affecting the reaction components and the reaction environment that were investigated in this experiment. **B** The comparison of the optimized *P. pastoris* CFPS system with the initial one. Based on the quantitative analysis (Additional file 1: Fig. S4), the sfGFP protein yield of the optimized system could reach 0.3 mg/mL

Therefore, a functional CFPS system of *P. pastoris* has been constructed (Fig. 6A). RI inhibited the degradation of mRNA. IRES and Kozak promoted the initiation of translation. Reaction time, extract amount, energy regeneration, amino acids, NTPs mixture, and metal ions all showed apparent effects on the protein expression. The results in Fig. 6B showed that the optimized system was significantly better than the initial system. In the exploration of RNase inhibitor addition, cell extract volume, reaction time, energy regeneration system, amino acids, NTPs, Mg^2+^ and K^+^, RNase inhibitor addition was the most influential factor for the CFPS system.

## Conclusion

Cell-free protein synthesis has developed into a powerful tool for synthetic biology, which can be used efficiently in many ways by better expanding and developing the eukaryotic CFPS system. In this study, we developed an efficient and easy-to-use yeast CFPS system derived from *P. pastoris* SMD1163 strain. In particular, three aspects of transcription, translation, and reaction conditions were screened or optimized to achieve efficient protein expression. The optimal translation initiation sequence CRPV + Kozak was gained. Optimization at the transcriptional level was achieved by adding RI to inhibit RNA degradation. A reaction time of 6 h was determined, as well as optimal reaction conditions of appropriate cell extract, CP/CPK, K^+^, and Mg^2+^. In this way, an efficient platform of *P. pastoris* CFPS based on IRES has been successfully built. To further improve the protein synthesis ability of *P. pastoris* CFPS system, in addition to the optimization of the CFPS system, the strain mutations, the extract preparation, and the addition of translation factors need to be explored deeply and broadly. In the future, it is hoped to further expand the potential scope of the platform by demonstrating the synthesis of different functional proteins. Using this system as a highly adaptive prototyping and synthesis platform, it can be used in more basic and applied research.

## Materials and methods

### Strains and media

The experimental strains were *Pichia pastoris* SMD1163 (*his4 pep4 prb1*) strain (Additional file 1: Table S1). SMD1163 strain was cultured in fresh YPD medium (1% yeast extract, 2% peptone, 2% glucose). The strain with plasmid was cultured in fresh LB medium (0.5% yeast extract, 1% tryptone, 1% NaCl). The RNase inhibitor used in this study was purchased from the company (Beyotime, China). RI addition was 20 U per individual reaction system.

### Plasmid construction

All plasmids were codon-optimized with the sfGFP gene sequence, replacing the original gene sequence between sites NdeI and SalI. Then, a 50-bp poly(A) tail was inserted into the 3' end of the sfGFP gene. The plasmid was cloned by Gibson DNA assembly method (Gibson et al. 2009). 14 IRES sequences were synthesized (Additional file 1: Table S2), and cloned into the vector before the sfGFP gene (Additional file 1: Figs. S1, 2). In addition, a Kozak sequence (GAAACG) was added after IRES. All constructs were verified by DNA sequencing (GENEWIZ, Suzhou, China) and extracted by gravimetric column purification method.

### Strain culture and extract preparation

The SMD1163 strain was cultured overnight at 30 ℃, 250 rpm in 5 mL of liquid YPD medium. Then, 250 mL of fresh YPD medium was inoculated with OD_600_ of 0.05. Cells were grown to an OD_600_ of 5 at 30 ℃, 250 rpm. Cells were collected by centrifugation at 4 ℃ for 15 min. Cells were washed three times with cold wash buffer (30 mM HEPES, pH 7.4, 100 mM potassium acetate, 2 mM magnesium acetate, 2 mM dithiothreitol). After the last wash and centrifugation, cells were weighed and resuspended in 1.5 mL of cold lysis buffer (30 mM HEPES, pH 7.4, 100 mM potassium acetate, 2 mM magnesium acetate, 2 mM dithiothreitol, 0.5 mM PMSF) per gram of cells. Lysed the cells twice using at 1000 bar pressure, centrifuged lysates at 4 ℃, 30,000 *g* for 30 min, transferred the supernatant to a new centrifuge tube, and centrifuged again under the same conditions. Carefully removed the supernatant and dialyzed it with a 3.5 kDa molecular weight cutoff (MWCO) membrane for buffer exchange. Four exchanges were performed with 50 volumes of fresh lysis buffer (30 mM HEPES, pH7.4, 100 mM potassium acetate, 2 mM magnesium acetate, 2 mM dithiothreitol, 0.5 mM PMSF), each dialyzed for 30 min at 4 ℃. After dialysis, they were centrifuged at 4 ℃, 21,000*g* for 30 min. The supernatant was collected, frozen in liquid nitrogen, and finally stored at − 80 ℃ (Additional file 1: Fig. S3).

### Cell-free protein synthesis

The standard CFPS reaction was carried out in a 1.5 mL microcentrifuge tube at 30 ℃. Each reaction (15 μL) contained the following components (Additional file 1: Table S3): 25 mM HEPES–KOH, pH 7.4, 120 mM potassium glutamate (MACKLIN, China), 4 mM magnesium glutamate (MACKLIN, China), 1.5 mM NTPs, 0.1 mM 20AA, 40 mM creatine phosphokinase (CP, from rabbit muscle; Sigma-Aldrich), 1.7 mM DTT (Beyotime, China), 0.6 mg/mL creatine phosphokinase (CPK, from rabbit muscle; Sigma-Aldrich), 16.7 µg/mL plasmid, T7 RNA polymerase, and 50% (v/v) cell extract. In general, the reaction occurred for 5 h.

### sfGFP quantitative expression

After the cell-free reaction, 2 µL reaction sample was mixed with 198 µL ddH_2_O and added into a black opaque 96-well plate. sfGFP fluorescence was measured using a microplate reader. The standard quantitative curve of sfGFP could represent the protein expression level (Additional file 1: Fig. S4). The excitation wavelength and emission wavelength of sfGFP were 485 nm and 528 nm, respectively. At least three independent experiments were performed.

### Supplementary Information

**Additional file 1: Fig. S1** Full PJL1-Crpv-Kozak-sfGFP sequence. **Fig. S2 **PCR products of 14 IRES analyzed by agarose gel electrophoresis. **Fig. S3 **Flow chart of the preparation of cell extracts. **Fig. S4 **Standard quantitative curve of sfGFP based on the fluorescence value and sfGFP protein expression level. **Table S1** Information of strain used as cell extract. **Table S2 **Information of IRES used in this study. **Table S3** Information of reagents.

## Data Availability

All data and materials are available in the manuscript and supporting information.

## Abbreviations

CFPS: Cell-free protein synthesis
IRES: Internal ribosomal entry site
ITAFs: IRES trans-acting factor
sfGFP: Super folder green fluorescent protein
CRPV: Cricket paralysis virus
RI: RNA enzyme inhibitor
CP: Phosphocreatine
CPK: Creatine phosphokinase
20AA: 20 Amino acid
