# Supplementary material for: IRES-mediated Pichia pastoris cell-free protein synthesis
Source: Bioresour Bioprocess. 2023 Jun 20;10(1):35. doi: 10.1186/s40643-023-00653-4 (PMC10992869; doi:10.1186/s40643-023-00653-4)
Supplement: Supplementary file 1 — Additional file 1: Fig. S1 Full PJL1-Crpv-Kozak-sfGFP sequence. Fig. S2 PCR products of 14 IRES analyzed by agarose gel electrophoresis. Fig. S3 Flow chart of the preparation of cell extracts. Fig. S4 Standard quantitative curve of sfGFP based on the fluorescence value and sfGFP protein expression level. Table S1 Information of strain used as cell extract. Table S2 Information of IRES used in this study. Table S3 Information of reagents. [file 40643_2023_653_MOESM1_ESM.docx]

**Additional file**

**IRES-mediated *Pichia pastoris* cell-free protein synthesis**

Yanan Wang^#^, Ting Wang^#^, Xinjie Chen, Yuan Lu*

Key Laboratory of Industrial Biocatalysis, Ministry of Education, Tsinghua University, Beijing 100084, China

Department of Chemical Engineering, Tsinghua University, Beijing 100084, China

# These authors contributed equally.

* Corresponding authors. Email: yuanlu@tsinghua.edu.cn (Y. Lu)


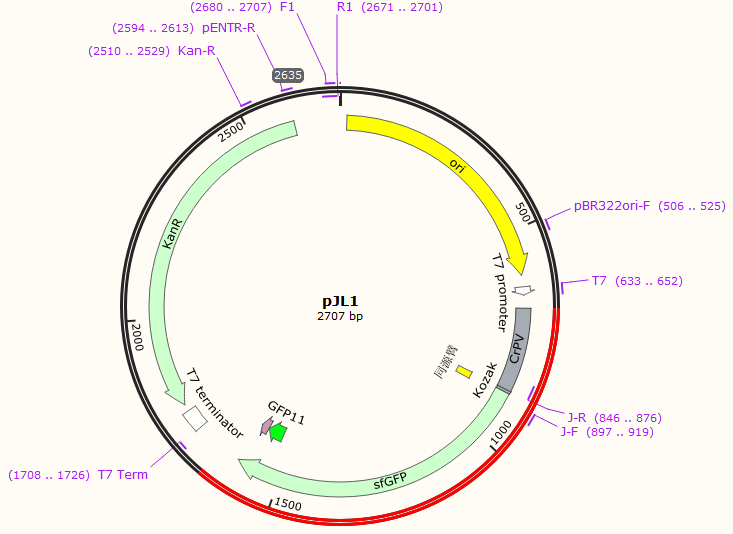


agatcaaaggatcttcttgagatcctttttttctgcgcgtaatctgctgcttgcaaacaaaaaaaccaccgctaccagcggtggtttgtttgccggatcaagagctaccaactctttttccgaaggtaactggcttcagcagagcgcagataccaaatactgttcttctagtgtagccgtagttaggccaccacttcaagaactctgtagcaccgcctacatacctcgctctgctaatcctgttaccagtggctgctgccagtggcgataagtcgtgtcttaccgggttggactcaagacgatagttaccggataaggcgcagcggtcgggctgaacggggggttcgtgcacacagcccagcttggagcgaacgacctacaccgaactgagatacctacagcgtgagctatgagaaagcgccacgcttcccgaagggagaaaggcggacaggtatccggtaagcggcagggtcggaacaggagagcgcacgagggagcttccagggggaaacgcctggtatctttatagtcctgtcgggtttcgccacctctgacttgagcgtcgatttttgtgatgctcgtcaggggggcggagcctatggaaaaacgccagcaacgcgatcccgcgaaattaatacgactcactatagggagaccacaacggtttccctctagaaataatAAAGCAAAAATGTGATCTTGCTTGTAAATACAATTTTGAGAGGTTAATAAATTACAAGTAGTGCTATTTTTGTATTTAGGTTAGCTATTTAGCTTTACGTTCCAGGATGCCTAGTGGCAGCCCCACAATATCCAGGAAGCCCTCTCTGCGGTTTTTCAGATTAGGTAGTCGAAAAACCTAAGAAATTTACCTGCTGAAACGatgTCTAAGGGAGAGGAGTTGTTCACCGGTGTTGTCCCAATCTTGGTTGAGTTGGACGGTGACGTTAACGGTCACAAGTTCTCCGTCAGAGGTGAGGGTGAAGGTGACGCCACCATCGGTAAGTTGACCTTGAAGTTTATCTGTACCACTGGTAAGTTGCCCGTTCCATGGCCAACCTTGGTTACCACTTTGACCTACGGTGTCCAGTGCTTCTCCAGATACCCAGACCACATGAAGCGTCACGATTTCTTCAAGTCCGCCATGCCAGAGGGTTACGTTCAAGAAAGAACTATTTCTTTCAAGGACGATGGTAAGTATAAAACCAGAGCTGTTGTCAAGTTCGAGGGTGACACCTTGGTCAACAGAATCGAGTTGAAGGGTACTGATTTTAAGGAAGATGGAAACATTTTGGGTCATAAGTTGGAATATAACTTCAACTCCCATAACGTCTACATCACCGCCGACAAGCAGAAGAACGGTATCAAGGCCAACTTCACCGTTCGTCACAACGTTGAGGATGGTTCCGTTCAGTTGGCTGACCACTACCAACAGAACACCCCAATTGGTGACGGACCAGTCTTGTTGCCAGACAACCACTACTTGTCCACCCAAACCGTCTTGTCCAAGGACCCAAACGAGAAGGGTACCAGAGACCACATGGTCTTGCACGAATACGTCAACGCTGCCGGAATCACTTGGTCTCATCCACAATTCGAAAAGTAAAAAAAAAAAAAAAAAAAAAAAAAAAAAAAAAAAAAAAAAAAAAAAAAAAAgtcgaccggctgctaacaaagcccgaaaggaagctgagttggctgctgccaccgctgagcaataactagcataaccccttggggcctctaaacgggtcttgaggggttttttgctgaaagccaattctgattagaaaaactcatcgagcatcaaatgaaactgcaatttattcatatcaggattatcaataccatatttttgaaaaagccgtttctgtaatgaaggagaaaactcaccgaggcagttccataggatggcaagatcctggtatcggtctgcgattccgactcgtccaacatcaatacaacctattaatttcccctcgtcaaaaataaggttatcaagtgagaaatcaccatgagtgacgactgaatccggtgagaatggcaaaagcttatgcatttctttccagacttgttcaacaggccagccattacgctcgtcatcaaaatcactcgcatcaaccaaaccgttattcattcgtgattgcgcctgagcgagacgaaatacgcgatcgctgttaaaaggacaattacaaacaggaatcgaatgcaaccggcgcaggaacactgccagcgcatcaacaatattttcacctgaatcaggatattcttctaatacctggaatgctgttttcccggggatcgcagtggtgagtaaccatgcatcatcaggagtacggataaaatgcttgatggtcggaagaggcataaattccgtcagccagtttagtctgaccatctcatctgtaacatcattggcaacgctacctttgccatgtttcagaaacaactctggcgcatcgggcttcccatacaatcgatagattgtcgcacctgattgcccgacattatcgcgagcccatttatacccatataaatcagcatccatgttggaatttaatcgcggcttcgagcaagacgtttcccgttgaatatggctcataacaccccttgtattactgtttatgtaagcagacagttttattgttcatgatgatatatttttatcttgtgcaatgtaacatcagagattttgagacacaacgtg

**Fig. S1** The full PJL1-CRPV-Kozak-sfGFP sequence. The sequence of sfGFP was highlighted in green, while the CRPV and Kozak were highlighted in yellow and light gray separately.

**
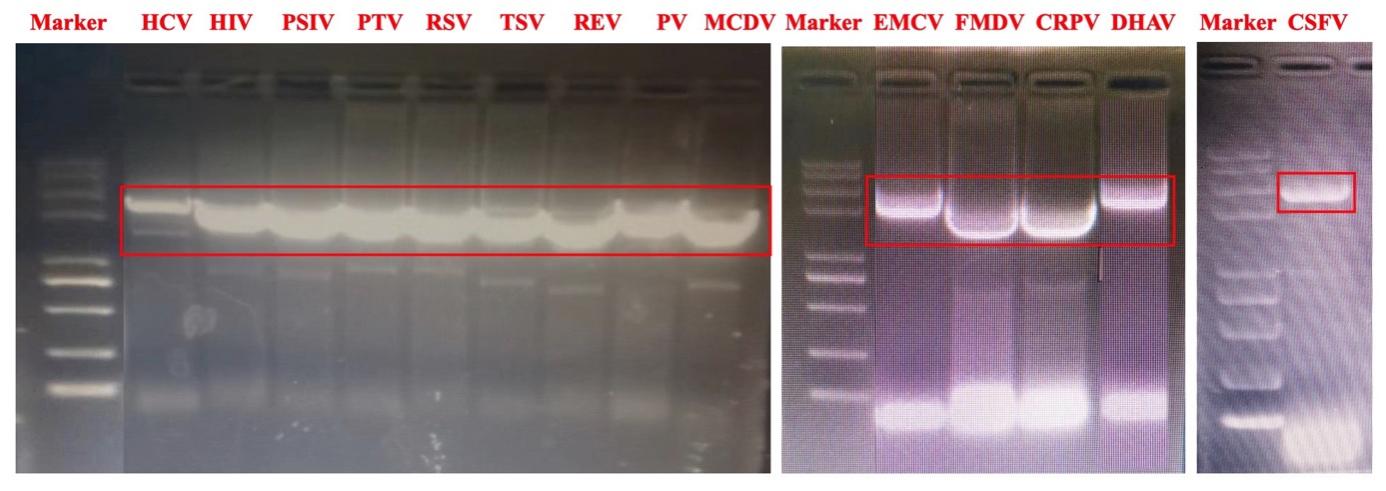
**

# Fig. S2 PCR products of 14 IRES analyzed by agarose gel electrophoresis. The red boxed area was the PCR product.


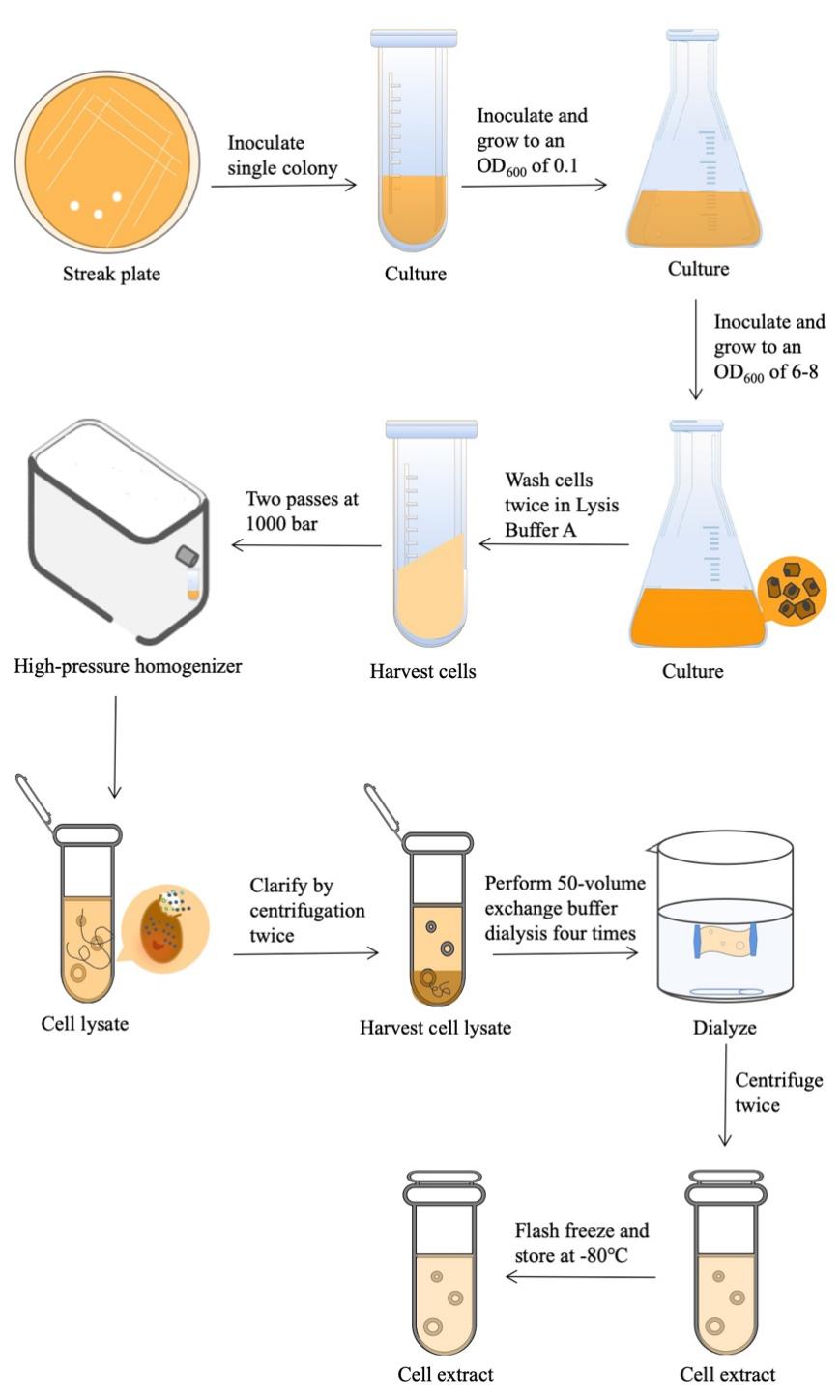


# Fig. S3 Flow chart of the preparation of cell extracts.


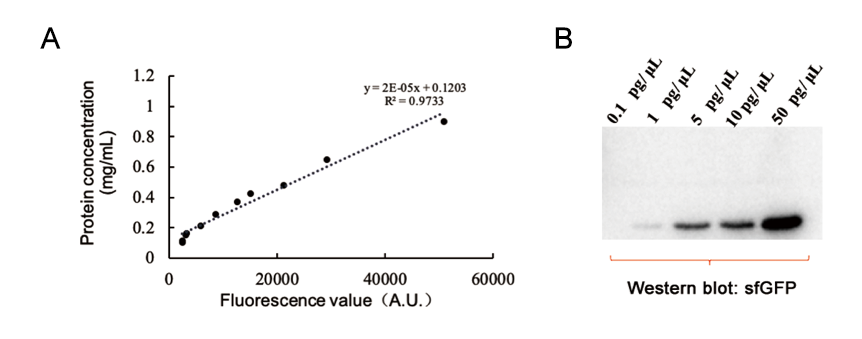


# Fig. S4 The standard quantitative curve of sfGFP based on the fluorescence value and sfGFP protein expression level.

# Table S1 Information of strain used as cell extract.

| Name | Characteristic |
| --- | --- |
| *Pichia pastoris* SMD1163 | Mutations in the *pep4* and *prb1* genes |

# Table S2 Information of IRES used in this study.

| Name | Sequence |
| --- | --- |
| Cricket paralysis virus (CRPV) | AAAGCAAAAATGTGATCTTGCTTGTAAATACAATTTTGAGAGGTTAATAAATTACAAGTAGTGCTATTTTTGTATTTAGGTTAGCTATTTAGCTTTACGTTCCAGGATGCCTAGTGGCAGCCCCACAATATCCAGGAAGCCCTCTCTGCGGTTTTTCAGATTAGGTAGTCGAAAAACCTAAGAAATTTACCTGCT |
| Poliovirus (PV) | TTAAAACAGCTCTGGGGTTGTACCCACCCCAGAGGCCCACGTGGCGGCTAGTACTCCGGTATTGCGGTACCCTTGTACGCCTGTTTTATACTCCCTTCCCGTAACTTAGACGCACAAAACCAAGTTCAATAGAAGGGGGTACAAACCAGTACCACCACGAACAAGCACTTCTGTTTCCCCGGTGATGTCGTATAGACTGCTTGCGTGGTTGAAAGCGACGGATCCGTTATCCGCTTATGTACTTCGAGAAGCCCAGTACCACCTCGGAATCTTCGATGCGTTGCGCTCAGCACTCAACCCCAGAGTGTAGCTTAGGCTGATGAGTCTGGACATCCCTCACCGGTGACGGTGGTCCAGGCTGCGTTGGCGGCCTACCTATGGCTAACGCCATGGGACGCTAGTTGTGAACAAGGTGTGAAGAGCCTATTGAGCTACATAAGAATCCTCCGGCCCCTGAATGCGGCTAATCCCAACCTCGGAGCAGGTGGTCACAAACCAGTGATTGGCCTGTCGTAACGCGCAAGTCCGTGGCGGAACCGACTACTTTGGGTGTCCGTGTTTCCTTTTATTTTATTGTGGCTGCTTATGGTGACAATCACAGATTGTTATCATAAAGCGAATTGGATTGGCCATCCGGTGAAAGTGAGACTCATTATCTATCTGTTTGCTGGATCCGCTCCATTGAGTGTGTTTACTCTAAGTACAATTTCAACAGTTATTTCAATCAGACAATTGTATCATA |
| Hepatitis C virus (HCV) | GCCTCCAGGACCCCCCCTCCCGGGAGAGCCATAGTGGTCTGCGGAACCGGTGAGTACACCGGAATTGCCAGGACGACCGGGTCCTTTCTTGGATAAACCCGCTCAATGCCTGGAGATTTGGGCGTGCCCCCGCAAGACTGCTAGCCGAGTAGTGTTGGGTCGCGAAAGGCCTTGTGGTACTGCCTGATAGGGTGCTTGCGAGTGCCCCGGGAGGTCTCGTAGACCGTGCACC |
| Classical swine fever virus (CSFV) | GTATACGAGGTTAGTTCATTCTCGTATACACGATTGGACAAATCAAAATTATAATTTGGTTCAGGGCCTCCCTCCAGCGACGGCCGAACTGGGCTAGCCATGCCCATAGTAGGACTAGCAAACGGAGGGACTAGCCGTAGTGGCGAGCTCCCTGGGTGGTCTAAGTCCTGAGTACAGGACAGTCGTCAGTAGTTCGACGTGAGCAGAAGCCCACCTCGAGATGCTACGTGGACGAGGGCATGCCCAAGACACACCTTAACCCTAGCGGGGGTCGCTAGGGTGAAATCACACCACGTGATGGGAGTACGACCTGATAGGGCGCTGCAGAGGCCCACTATTAGGCTAGTATAAAAATCTCTGCTGTACATGGCAC |
| Plautia stali intestine virus (PSIV) | GACTATGTGATCTTATTAAAATTAGGTTAAATTTCGAGGTTAAAAATAGTTTTAATATTGCTATAGTCTTAGAGGTCTTGTATATTTATACTTACCACACAAGATGGACCGGAGCAGCCCTCCAATATCTAGTGTACCCTCGTGCTCGCTCAAACATTAAGTGGTGTTGTGCGAAAAGAATCTCACTTCAA |
| Encephalomyocarditis virus (EMCV) | TTGCCAGTCTGCTCGATATCGCAGGCTGGGTCCGTGACTACCCACTCCCCCTTTCAACGTGAAGGCTACGATAGTGCCAGGGCGGGTACTGCCGTAAGTGCCACCCCAAACAACAACAACAAAACAAACTCCCCCTCCCCCCCCTTACTATACTGGCCGAAGCCACTTGGAATAAGGCCGGTGTGCGTTTGTCTACATGCTATTTTCTACCGCATTACCGTCTTATGGTAATGTGAGGGTCCAGAACCTGACCCTGTCTTCTTGACGAACACTCCTAGGGGTCTTTCCCCTCTCGACAAAGGAGTGTAAGGTCTGTTGAATGTCGTGAAGGAAGCAGTTCCTCTGGAAGCTTCTTAAAGACAAACAACGTCTGTAGCGACCCTTTGCAGGCAGCGGAACCCCCCACCTGGTGACAGGTGCCTCTGCGGCCAAAAGCCACGTGTATAAGATACACCTGCAAAGGCGGCACAACCCCAGTGCCACGTTGTGAGTTGGATAGTTGTGGAAAGAGTCAAATGGCTCTCCTCAAGCGTATTCAACAAGGGGCTGAAGGATGCCCAGAAGGTACCCCATTGTATGGGATCTGATCTGGGGCCTCGGTGCACGTGCTTTACACGTGTTGAGTCGAGGTGAAAAAACGTCTAGGCCCCCCGAACCACGGGGACGTGGTTTTCCTTTGAAAACCACGATTACAAT |
| Porcine teschovirus (PTV) | ACTTGGTTATGAATTCATTGTATTAACCCCTCTGAAAGACCTGCTCTGGCGCGAGCTAAAGCGCAATTGTCACCAGGTATTGCACCAATGGTGGCGACAGGGTACAGAAGAGCAAGTACTCCTGACTGGGTAATGGGACTGCATTGCATATCCCTAGGCACCTATTGAGATTTCTCTGGGGCCCACCAGCGTGGAGTTCCTGTATGGGAATGCAGGACTGGACTTGTGCTGCCTGACAGGGTCGCGGCTGGCCGTCTGTACTTTGTATAGTCAGTTGAAA |
| Taura syndrome virus (TSV) | TAGCACCACCCGATCGTAAACTCCATGTATTGGTTACCCATCTGCATCGAAAACTCTCCGAACACTAGGTGCAGTAAGGCTTTCATGGAGTGGTTTGCTATTTAGCGTACGTGTACCATAGGCAGCCCCAAAAACACGTGTGAGGAGAAAGTCCCAGTCACTTTGGGCAAAGTAGACAGCCGCGCTTGCGTGGTGGGACTTAATTAATGCCT |
| Reticuloendotheliosis virus (REV) | CGACAGCGCCCTGCGAATTGGTGTGCCCACACCGCGCGGCTTGCGAATAATACTTTGGAGAGTCTTTTGCCTCCAGTGTCTTCCGTTTGTACTCGTCCTCCTCTCCCTCTCCGGCCGGGATGGGACAGG |
| Duck hepatitis A virus (DHAV) | TAGTGGTTAGCCTACCACCCCTTGGCCACTAATTCTTGGCTTTCTGTTTTGGGATCCACCATATCTTGGAGGTGGTGCTGAAATATTGCAAGCCACATGGTATCTGTGTGTTTTCTAAACATGAAGCTTTGGTTCAGTGGTTTTGGACAAGGAAAGGCTAGTGTTTGGTCTGGGTACAAACCCTTGTTGTGAAACGGATTACCGGTAGTAGCATCTAGTGGTTCCAGTCCATAACATGAGTGTATGGTCTAGAGTGGACATAGCTTGGATACAGACACCTTCAGTATTACTGGGTGTTCCAGACTAGTTCCTGAGGTACCAAGTTATGAGGGGCTATGGGAAAACCCCTTTGATCCACACTGCCTGATAGGGTCGCGGCTGGTCGAGTCCCATACACTATAAAACCAGTTGACTTTCATGCA |
| Foot-and-mouth disease virus (FMDV) | CTTGGTTTCTGGAGACAGGCTAAGGATGCCCTTCAGGTACCCCGAGGTAACAAGCGACACTCGGGATCTGAGAAGGGGATTGGGGCTTCTATCCAAGCGCCCAATTTAAAAAGCTTCTATGCCTGAATAGGTGACCGGAGGCCGGCACCTTTTCCTTTACCCACAACTTACTTT |
| Human immunodeficiencyvirus  (HIV) | TGTGTGCCCGTCTGTTGTGTGACTCTGGTAACTAGAGATCCCTCAGACCCTTTTAGTCAGTGTGGAAAATCTCTAGCAGTGGCGCCCGAACAGGGACCTGAAAGCGAAAGGGAAACCAGAGGAGCTCTCTCGACGCAGGACTCGGCTTGCTGAAGCGCGCACGGCAAGAGGCGAGGGGCGGCGACTGGTGAGTACGCCAAAAATTTTGACTAGCGGAGGCTAGAAGGAGAGAG |
| Rous sarcoma virus (RSV) | CTCGGTTGTAACGCGCTTAGGAAGTCCCCTCGAGGTATGGCAGATATGCTCTTGCATAGGGGGAAAAAATGTAGTCTTAATATTGTCTGTGTGCTGCAGGAGCTAAGCTGACTCTGCTGGTGGCCTCGCGTACCACTGTGGCCAGGCGGTAGCTGGGACGTGCAGCCGACCACC |
| Mud crab dicistrovirus (MCDV) | CATTTATTTATATTAAATACTGACACTTTGCGGGTTAAAATGTTTAATACTATTTTTCAATTTGAGGTTGTATGAGATAATTTTGATTTCTATGTTATCATTGAATAAGGGAGTCTGGCCCTAATTGATGTACGACTCTTCTTTGGTTGCGACCCGAGTCCCTTCTACATCAAG |

# Table S3 Information of reagents.

| Name | Composition |
| --- | --- |
| HEPES-KOH | 25 mM HEPES; Using KOH to adjust pH to 7.4 |
| NTPs | 25 mM spermidine; 1.5 mM folinic acid; 37.5 mM ATP; 37.5 mM GTP; 22.5 mM CTP; 22.5 mM UTP; 8.25 mM NAD; 6.8 mM CoA; 18.75 mM cAMP; 5 mg/mL tRNA |
| 20AA | 50 mM arginine; 50 mM valine; 50 mM tryptophan; 50 mM phenylalanine; 50 mM isoleucine; 50 mM leucine; 50 mM cysteine; 50 mM methionine; 50 mM alanine; 50 mM aspartic acid; 50 mM aspartic acid; 50 mM glycine; 50 mM glutamine; 50 mM lysine; 50 mM proline; 50 mM serine; 50 mM threonine; 50 mM tyrosine; 50 mM glutamine; 50 mM histidine; Using HCl to adjust pH to 7.4 |
